# One-Step In Situ Self-Assembly of Cypress Leaf-Like Cu(OH)_2_ Nanostructure/Graphene Nanosheets Composite with Excellent Cycling Stability for Supercapacitors

**DOI:** 10.1186/s11671-019-3000-4

**Published:** 2019-05-17

**Authors:** Zhihao Zhai, Yuxiu You, Liguo Ma, Dongkai Jiang, Fanggang Li, Hao Yuan, Maojun Zheng, Wenzhong Shen

**Affiliations:** 1 Key Laboratory of Artificial Structures and Quantum Control (Ministry of Education), School of Physics and Astronomy, Shanghai Jiao Tong University, Shanghai, China; 2 Collaborative Innovation Center of Advanced Microstructures, Nanjing University, Nanjing, China

**Keywords:** Cypress leaf-like Cu(OH)_2_ nanostructure, graphene nanosheets, outstanding cycling performance

## Abstract

Transition metal hydroxides and graphene composite holds great promise to be the next generation of high performance electrode material for energy storage applications. Here we fabricate the cypress leaf-like Cu(OH)_2_ nanostructure/graphene nanosheets composite through one-step in situ synthesis process, employed as a new type of electrode material for high efficiency electrochemical energy storage in supercapacitors. A solution-based two-electrode system is applied to synthesize Cu(OH)_2_/graphene hybrid nanostructure, where anodic graphene nanosheets firmly anchor cathodic Cu(OH)_2_ nanostructure due to the electrostatic interaction. The in situ self-assembly of Cu(OH)_2_/graphene ensures good structural robustness and the cypress leaf-like Cu(OH)_2_ nanostructure prompt to form the open and porous morphology. The hybrid structure would facilitate charge transport and effectively mitigate the volume changes during long-term charging/discharging cycles. As a consequence, the Cu(OH)_2_/graphene composite exhibits the highest capacitance of 317 mF/cm^2^ at the current density of 1 mA/cm^2^ and superior cyclic stability with no capacitance decay over 20,000 cycles and remarkable rate capability at increased current densities.

## Introduction

The ever depletion of fossil fuels and aggravation of environmental pollutions call for urgently exploring sustainable energy sources and developing energy storage technologies to meet application requirements of many electronic devices and hybrid vehicles in our modern society [1, 2]. As a promising energy storage device, supercapacitors (SCs) have attracted much attention in view of their small size, high power density, fast recharge ability, long lifespan and desirable operational safety [3–8] There are two classes of SCs, pseudocapacitors and electrical double layer capacitors (EDLCs), on the basis of energy storage mechanism [9]. Carbon material with many advantages of abundance, non-toxic, large surface area, good conductivity, excellent chemical durability, is a typical electrode material for double-layer capacitors (EDLCs), storing charge in the electric double-layer near electrolyte/electrode surface by electrostatic adsorption [10–16]. However, carbon material generally exhibits a relatively low specific capacitance. By comparison, many inexpensive transition metal hydroxides, such as Ni(OH)_2_ [17, 18], NiO [19], MnO_2_ [20], Co_3_O_4_ [21] store energy partially relied on fast reversible Faradic redox reactions occurring on the electrode surface, offering much higher pseudo-capacitance [22, 23]. Unfortunately, most of them suffer from the intrinsic poor electric conductivity and undergo huge volume change during electrochemical processes, which results in the poor reversibility and short cycle life [24]. Obviously, to synthesize the high-performance electrode material at a low cost, it is of great significance to combine easily available transition metal hydroxides with carbon material by a cost-effective and facile fabrication strategy.

Among various transition hydroxides, Cu(OH)_2_ is one of the most promising electrode material because of its natural abundance, environmentally friendly and fast redox couple [25–27]. Besides the above -mentioned characteristics of most carbon material, graphene has an exceptionally large specific surface area, whose major surfaces are exposed to the electrolyte, exhibiting a high specific capacitance (550 F/g) [28]. To improve the electric conductivity and enhance capacity of electrode, Cu(OH)_2_ and graphene composite have been designed as electrode, efficiently inhibiting the volume changes of Cu(OH)_2_ and preventing serious agglomeration and re-stacking of graphene because the typical flexible and robust nature of graphene enable electrode materials to effectively maintain the structural integration [26, 29–31]. Mahanty et al. presented that the reduced graphene oxide/Cu(OH)_2_ composite, which exhibited a high capacitance of 602 F g^−1^ and good capacitance retention of 88.8% over 5000 cycles. Both specific capacitance and cyclic stability were dramatically enhanced, compared with pristine Cu(OH)_2_ [26]. Ghasemi et al. prepared Cu_2_O-Cu(OH)_2_-graphene nanocomposite by multiple steps, including electrophoretic deposition and electrodeposition techniques, exhibited specific capacitance of 425 F g^−1^ and maintained about 85% of initial capacitance with a current density of 10 A g^−1^ after 2500 cycles [32]. Although supercapacitive properties have been enhanced in the report, most of these approaches are complicated and expensive. Furthermore, the cycling stability of reported Cu(OH)_2_/graphene composite for supercapacitance needs to be further improved.

In this work, we report the one-step in situ self-assembly of cypress leaf-like Cu(OH)_2_ nanostructure/graphene nanosheets composite realizes in a two-electrode system, where graphene nanosheets generate from electrochemical exfoliation of graphite at anode and simultaneously Cu(OH)_2_ nanostructure forms on Cu foam at cathode. The morphology and structure, together with the interaction between different components of nanocomposite would influence their electrochemical energy storage properties. The transparent few-layer graphene nanosheets firmly anchor on cypress leaf-like Cu(OH)_2_ surface, forming a porous, open and interconnected structure. This unique hybrid structure is expected to endow this composite fast charge transfer velocity, high electrochemical activity, and excellent stability. As a result, the Cu(OH)_2_/graphene composite presents excellent electrochemical energy storage performance with high specific capacitance and wonderful cyclic stability over 20,000 cycles, making it an ideal electrode material for high-performance SCs.

## Methods Section

### Sample Preparation

**Fig. 1:**
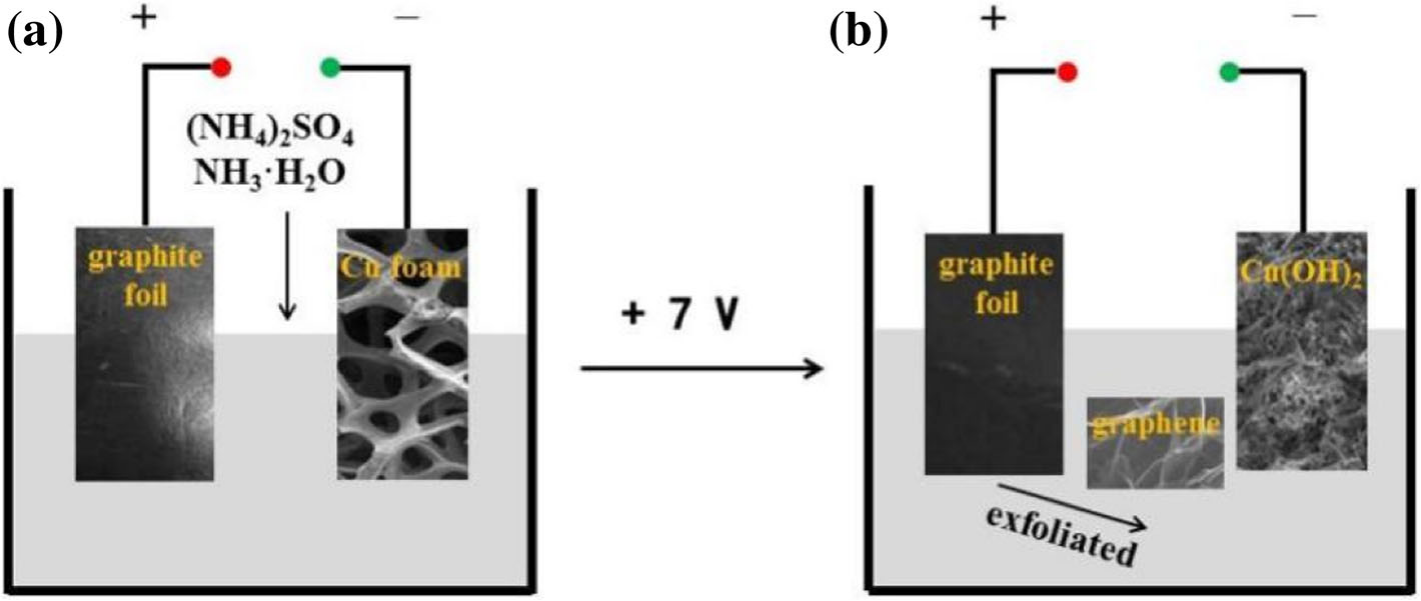
Schematic diagram of the experimental set-up of one-step in situ self-assembly of Cu(OH)_2_/graphene composite

The copper foam (10 × 15 × 1.6 mm^3^, Xiamen Yongchangshuo Electronic Technology Co. Ltd., China) and graphite foil (10 × 15 × 1.0 mm^3^, Shanghai Alfa Aesar Chemical Co. Ltd., China) slices were washed in an ultrasonic bath with absolute ethanol and DI water for 15 min respectively [33], afterward the slices were placed in deionized water for later use. As illustrated in Fig. 1, the electrochemical synthesis process was implemented in a two-electrode cell system [9], where graphite foil acts as anode and Cu foam acts as cathode. In order to achieve in situ self-assembly of cypress leaf-like Cu(OH)_2_ nanostructure/graphene nanosheets composite, the electrolyte is a mixed solution of 0.1 M (NH_4_)_2_SO_4_ (100 mL) and NH_3_·H_2_O (3 mL). When the two-electrode cell system was applied to a direct current voltage of 7 V for 1 h, at anode the graphite foil was electrochemically exfoliated and decomposed into a lot of graphene nanosheets and at cathode Cu foam was corroded into cypress leaf-like Cu(OH)_2_ by NH_3_·H_2_O.

1 $$ \mathrm{Cu}+6\ \mathrm{N}{\mathrm{H}}_3+2\ {\mathrm{H}}_2\mathrm{O}\to {\left[\mathrm{Cu}{\left(\mathrm{N}{\mathrm{H}}_3\right)}_6\right]}^{2+}+2\ \mathrm{O}{\mathrm{H}}^{-}+{\mathrm{H}}_2\uparrow $$

2 $$ \mathrm{C}{\mathrm{u}}^{2+}+2\ \mathrm{O}{\mathrm{H}}^{-}\to \mathrm{C}\mathrm{u}{\left(\mathrm{OH}\right)}_2 $$

Driven by the electric field, the exfoliated graphene nanosheets with residual negative charges at its edge were electrically attracted to the surface of cathodic Cu(OH)_2_, assembling into this unique porous nanostructure. The resulting cypress leaf-like Cu(OH)_2_ nanostructure/graphene nanosheets composite was air dried.

### Characterizations

The X-ray diffraction (XRD) was carried out on a Rigaku Ultima IV X-ray Diffractometer by Cu Kα radiation with the scan rate of 2°min^−1^ over a 2*θ* range from 10° to 80°. Raman spectroscopy was acquired on Renishaw in a Via-reflex system, with the excitation source of a laser wavelength (532 nm). We obtain the details of morphology, structure, crystal size, and other parameters by field emission scanning electron microscopy (FESEM, Zeiss Ultra Plus), transmission electron microscope (TEM), and selected-area electron diffraction (SAED) (JEOL JEM-2100F operating at 200 kV). The surface chemical components and valance states of the sample were researched by X-ray photoelectron spectroscopy (XPS).

### Electrochemical Measurements

The electrochemical measurements of the Cu(OH)_2_/graphene composite on Cu foam was implemented in a three-electrode configuration with a Ag/AgCl electrode as reference electrode and a Pt plate electrode as counter electrode in 1 M KOH electrolyte. The cyclic voltammetry (CV), and electrochemical impedance spectroscopy (EIS) tests were conducted on PARSTAT 4000. The CV curves and galvanostatic charge-discharge measurements (GCD) were carried out within the potential window from 0 V to 0.6 V, respectively. The GCD and cyclic stability were performed on LAND CT-2001A. The EIS was tested with no bias voltage with the frequency range of 0.01–100 kHz. The area capacitance of the sample was calculated by the following equation:

3 $$ C=\frac{Jt}{\Delta  V} $$

, in which *C* (mF cm^−2^) represents the area capacitance, *J* (mA cm^−2^) is the current density, *t* (*s*) is the discharging time, Δ*V* (*V*) is the voltage window for cycling tests.

## Results and Discussions

**Fig. 2:**
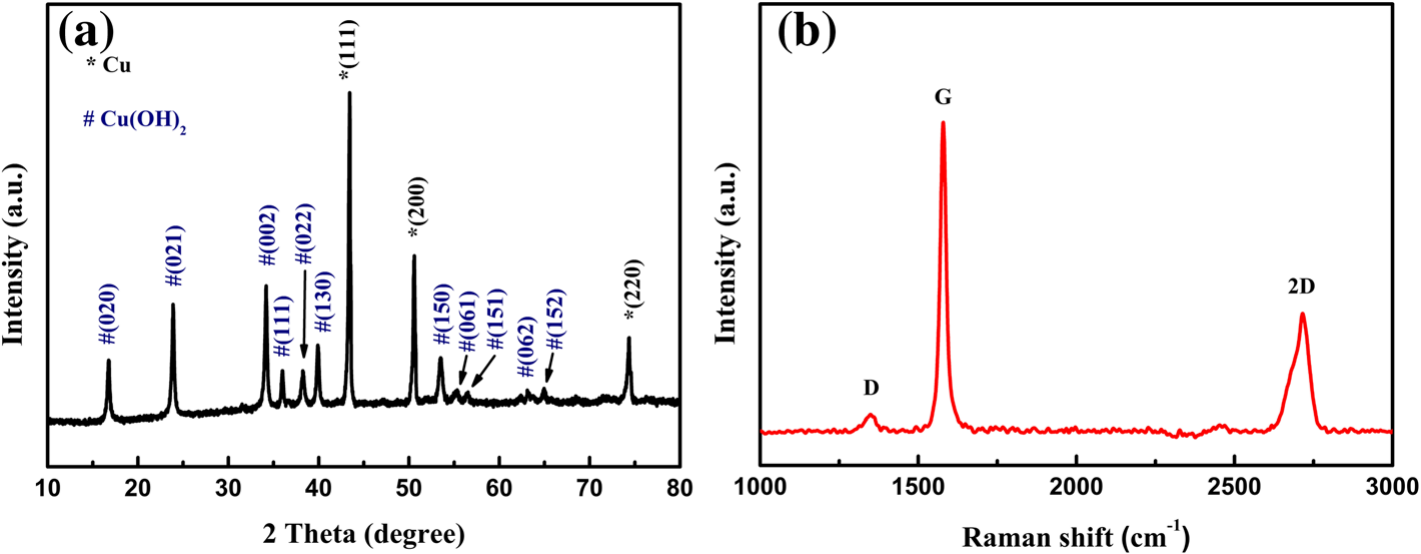
**a** X-ray diffractogram and **b** Raman spectra of Cu(OH)_2_/graphene composite

The formation and phase purity of Cu(OH)_2_/graphene composite were studied by X-ray diffraction (Fig. 2a). The peaks marked with an asterisk at 43.4^°^, 50.6^°^, and 74.4^°^ are corresponding to the metallic copper (JCPDS 04-0836) of the copper foam. While the diffraction peaks located at 16.7^°^, 23.9^°^, 34.2^°^, 36.0^°^, 38.3^°^, 39.9^°^, 53.5^°^, 55.3^°^, 56.5^°^, and 65.0^°^ are in good agreement with Cu(OH)_2_ (JCPDS 01-080-0656). The sharp peaks in the diffraction pattern indicate that the synthesis material has good crystallinity and high pure Cu(OH)_2_ phase. Raman spectroscopy is a significant instrument for characterization of carbon materials. Figure 2b shows the Raman spectrum for the Cu(OH)_2_/graphene composite. The Raman spectra exhibit three noticeable peaks at 1349 cm^−1^, 1579 cm^−1^, and 2715 cm^−1^ corresponding to the D-band, G-band, and 2D-band of graphene, respectively, which confirmed the existence of graphene [9].

**Fig. 3:**
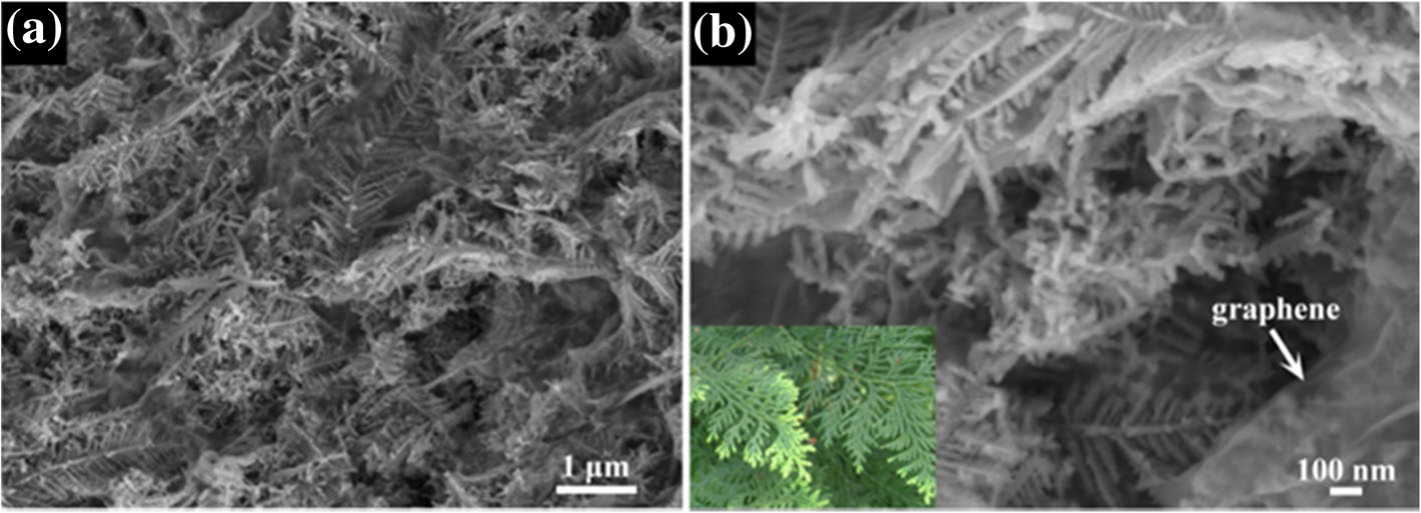
FESEM images of the Cu(OH)_2_/graphene composite at **a** low and **b** high magnification

Figure 3 displays the morphology and structure of cypress leaf-like Cu(OH)_2_ nanostructure/graphene nanosheets. As shown in Fig. 3a, a typical FESEM image shows that the Cu(OH)_2_ nanostructure interweave with the graphene nanosheets to form a highly open and porous interconnected nanostructure. Figure 3b presents the enlarged FESEM image of some representative Cu(OH)_2_/graphene composite and indicates that the in situ synthesized Cu(OH)_2_ composed of short one-dimensional nanorod has a similar morphology of cypress leaf and the graphene nanosheets are ultrathin and transparent. This Cu(OH)_2_/graphene hybrid nanostructure is expected to have a large surface area, good ion accessibility, and mechanical adhesion.

**Fig. 4:**
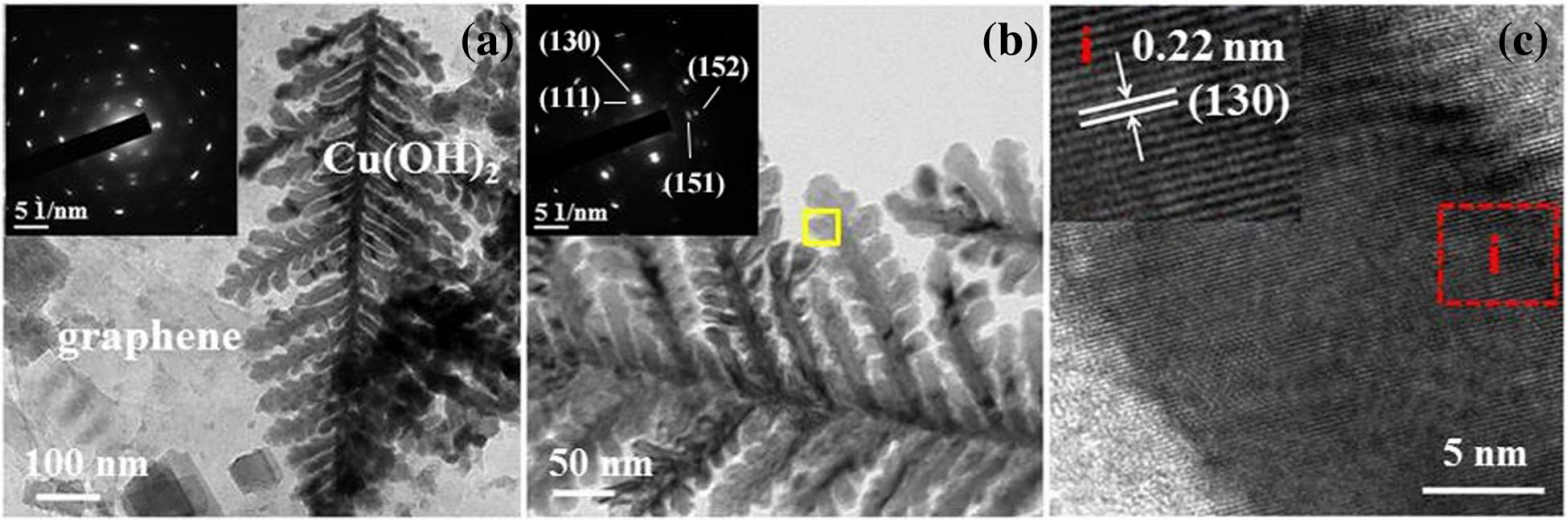
**a** TEM image of the Cu(OH)_2_/graphene composite. The inset SAED pattern originates from graphene nanosheets. **b** High-magnification TEM image with the SAED of one branch of cypress leaf-like Cu(OH)_2_ in the inset. **c** High-resolution TEM image of the marked area in Fig. 4b

The detailed nanostructure of the Cu(OH)_2_/graphene composite is analyzed by TEM. The low magnification TEM image in Fig. 4a shows that the cypress leaf-like Cu(OH)_2_ nanostructure attached to ultrathin graphene nanosheets, which be consistent with the SEM images. We performed selected area electron diffraction (SAED) of graphene as shown in the inset Fig. 4a. The well-defined diffraction spots and hexagonal diffraction pattern confirm the crystalline structure of the graphene nanosheets obtained via exfoliation from graphene foil. From the high-magnification TEM images (Fig. 4b), we can find the branches of cypress leaf-like Cu(OH)_2_ nanostructure have an average length of 300 nm and diameter of 15 nm. Moreover, the clearly visible diffraction spots in the SAED pattern (inset of Fig. 4b) reveals that the branch of cypress leaf-like Cu(OH)_2_ has a good crystallinity. The diffraction spots with a calculated d-spacing of 0.25 nm, 0.22 nm, 0.16 nm, and 0.14 nm can be associated with the (111), (130), (151), and (152) facet of Cu(OH)_2_. Figure 4c depicts a HRTEM image and the lattice fringe of 0.22 nm is assigned to (130) facet of Cu(OH)_2_. Observation of clear lattice fringe further confirms the formation of the branches of cypress leaf-like Cu(OH)_2_ with good crystallinity.

**Fig. 5:**
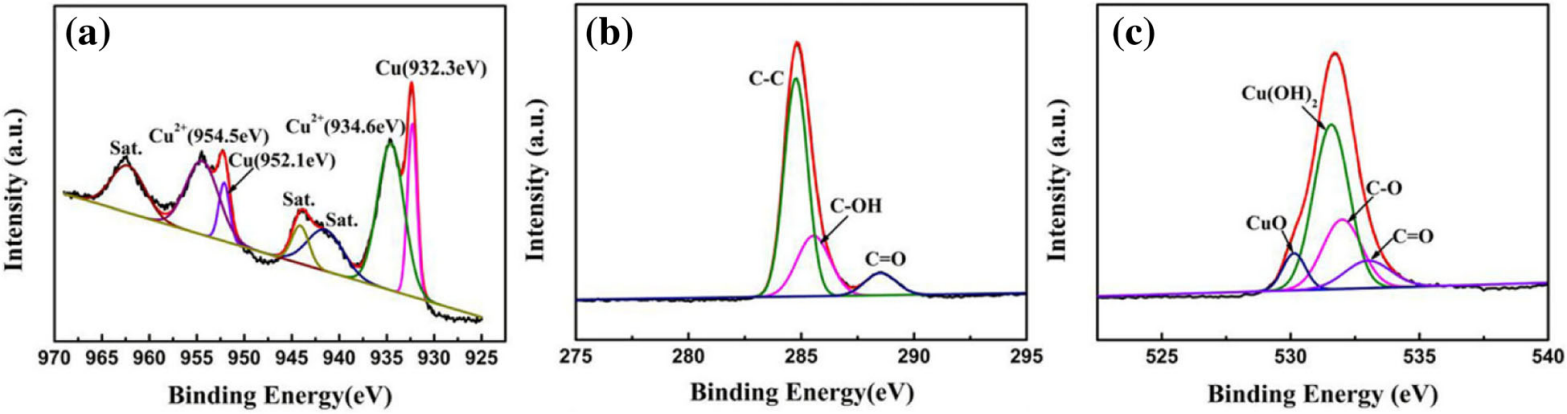
XPS spectra of **a** Cu 2p, **b** C 1s. and **c** O 1s

The chemical valence states and element composition are characterized by deconvoluted XPS spectra as presented in Fig. 5. The XPS of Cu 2p is displayed by Fig. 5a. The peak observed at 954.5 eV and 934.6 eV are indexed to Cu 2p_1/2_ and Cu 2p_3/2_ peaks of Cu^2+^, respectively, indicating the existence of Cu(OH)_2_. Due to the Cu foam as substrate, the characteristic peaks at 952.1 eV and 932.3 eV are from Cu 2p_1/2_ and Cu 2p_3/2_. The C 1s XPS spectrum (Fig. 5b) of Cu(OH)_2_/graphene is deconvoluted into three peaks: C=O (288.5 eV), C-OH (285.6 eV), and C-C (284.8 eV), respectively. O 1s spectra (Fig. 5c) has two contributions: the two peaks at 531.6 eV and 530.1 eV can be assigned to the oxygen species in Cu(OH)_2_ and CuO, respectively, the other two peaks at 532 eV and 533 eV originates from C-O and C=O, respectively.

**Fig. 6:**
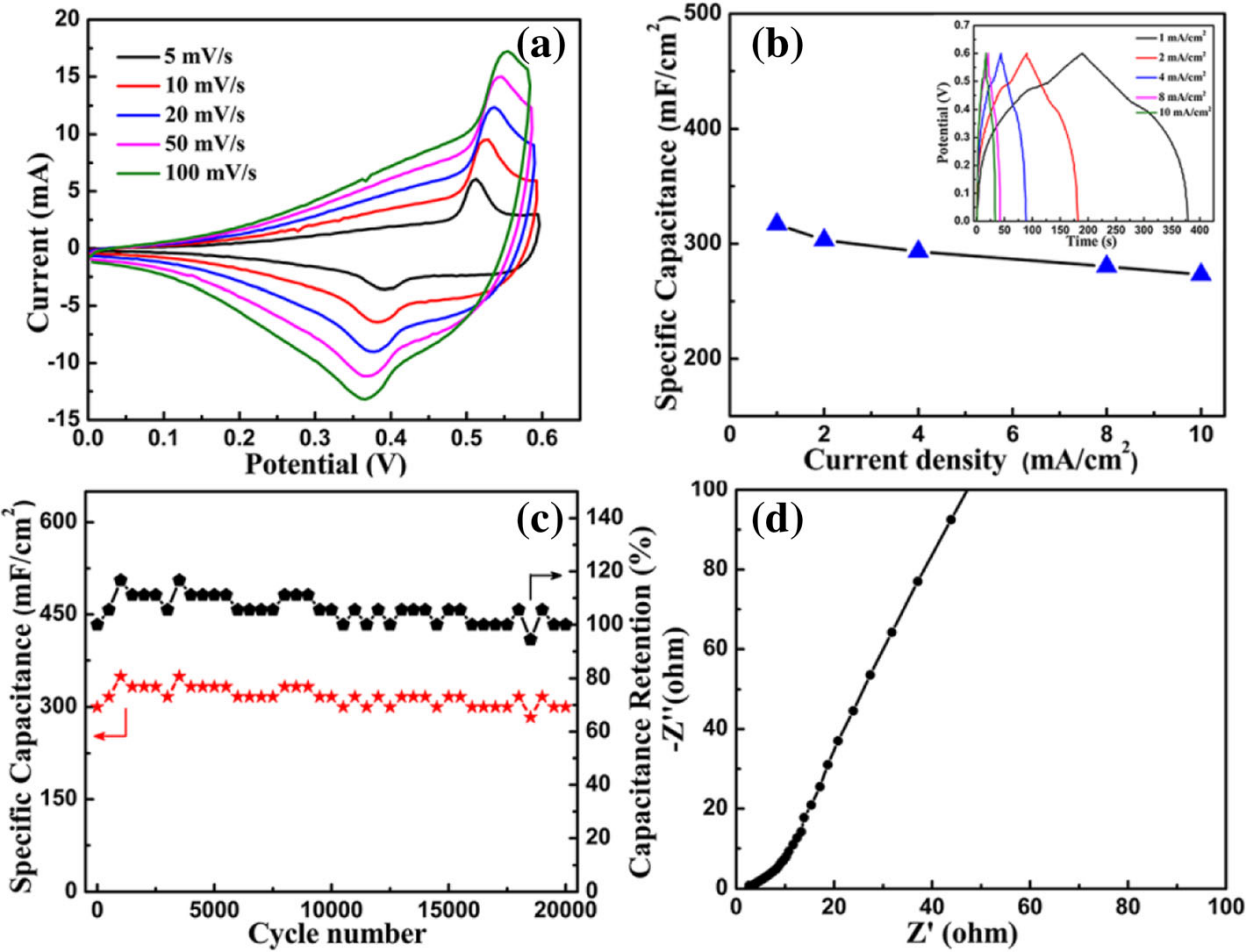
Electrochemical performance of the Cu(OH)_2_/graphene composite. **a** CV curves. **b** The specific capacitance and galvanostatic charging/discharging curves. **c** Area capacitance and Coulombic efficiency at a current density of 2 mA cm^−2^. **d** Nyquist plot of the Cu(OH)_2_/graphene

The electrochemical charge storage capability of the Cu(OH)_2_/graphene nanocomposite was investigated by taking them as working electrodes. The cyclic voltammogram (CV) curves of Cu(OH)_2_/graphene are shown in Fig. 6a, when tested at various scan rates with the range from 5 mV s^−1^ to 100 mV s^−1^. A pair of well-defined redox peaks is obviously observed in each curve, corresponding to the reversible reaction of Cu^2+^ ↔ Cu^1+^. The reversible redox reactions can be expressed as [27]

4 $$ 2\ \mathrm{Cu}{\left(\mathrm{OH}\right)}_2+2\ {\mathrm{e}}^{-}\kern0.5em \Longleftrightarrow \mathrm{C}{\mathrm{u}}_2\mathrm{O}\kern0.5em +2\ \mathrm{O}{\mathrm{H}}^{-}+{\mathrm{H}}_2\mathrm{O} $$

With increasing scan rate, CV curves maintain a similar profile and the current response increased, indicating the good rate capability and good reversibility of Faradic reactions [17, 27]. Meanwhile, the oxidation and reduction peak respectively shift to more positive and more negative potentials, due to the limited ion diffusion time or high-electron hopping resistance [34].

Figure 6b displays the area capacitance and galvanostatic charge-discharge curves at different current densities of 1, 2, 4, 8, and 10 mA cm^−2^. The galvanostatic charge-discharge curves of the composite electrode exhibit the typical pseudo-capacitive nature, which finely agrees with its CV curves. The Cu(OH)_2_/graphene composite achieves the highest area-specific capacitance of 317 mF cm^-2^ at a current density of 1 mA cm^-2^. The specific capacitance can maintain 303, 293, 280, 273 mF cm^−2^ at different current densities. The Cu(OH)_2_/graphene nanocomposite electrode shows a good rate capability with only 14% capacitance loss at a high current density of 10 mA cm^−2^, which can be ascribed to the unique nanostructure in favor of fast and efficient electrolyte ion diffusion and charge transfer [17].

The cycling stability of the Cu(OH)_2_/graphene nanocomposite electrode was studied by charging-discharging cycling measurements at the constant current density of 2 mA cm^−2^ (Fig. 6c). The specific capacitance till 20,000 cycles keep the initial value of 303 mF cm^−2^ with 100% retention, exhibiting the outstanding cycling performance. Moreover, the Coulombic efficiency can maintain 100% which further demonstrates that the electrode possesses good electrochemical stability. Form Fig. 6d, the intercept value about 2.35 on real axis represents the internal resistance (*R*_S_) in high-frequency area. The slightly high internal resistance is mainly attributed to the inherent resistance of active material, due to the natural defect in electric conductivity of Cu(OH)_2_. The slope of the Nyquist plot reflects the Warburg impedance, which demonstrates a low electrolyte diffusion resistance. The open porous Cu(OH)_2_/graphene nanocomposite nanostructure with large surface area endows the electrode with abundant reactive sites and shorten ion diffusion path.

The excellent electrochemical energy storage properties of the Cu(OH)_2_/graphene nanocomposite are ascribed to the following reasons: (i) the 3D Cu foam substrate analogous to the reported Ni foam also has many advantages of high-electric conductivity, large surface area, microscale pores and many flow channels, providing the active material with high mass loading, and large effective surface area [35, 36]; (ii) due to the cypress leaf-like Cu(OH)_2_ synthesized by in situ oxidation of Cu foam, this binder-free electrode not only reduce the dead volume effect and the internal resistance but also prompt the effective charge transfer and fast redox reactions [37, 38]; (iii) the electric conductivity of the Cu(OH)_2_ can be improved by assembling with graphene, facilitating the electrolyte ions diffusion and electron transport [39]; (iv) to some extent, the volume changes of Cu(OH)_2_ and especially the agglomeration of graphene all can be alleviated, increasing the stability of both nanostructure and electrochemical performance during continuous charge-discharge processes [29]; (v) the unique open, porous, and interconnected nanostructure can reserve electrolyte ions to ensure the sufficient redox reactions particularly at high current densities [40].

## Conclusions

We have adopted a simple electrochemical method based on solution to in situ synthesize cypress leaf-like Cu(OH)_2_ nanostructure/graphene nanosheets on Cu foam serving as a promising electrode for supercapacitors. This novel hybrid nanostructure endows the Cu(OH)_2_/graphene nanocomposite with abundant redox reactions, good charge transfer, and short electrolyte ion diffusion pathway. When evaluated as the electrode material for supercapacitors, the Cu(OH)_2_/graphene nanocomposite demonstrates high reversible capacitance of 317 mF cm^−2^ and excellent stability with 100 % retention over 20,000 cycles at current densities of 2 mA cm^−2^ and remarkable rate capability at increased current densities. This synthesis method will open a new door for the facile fabrication of other hydroxides and provides an effective strategy for remarkable electrochemical energy storage devices.

## Abbreviations

CV: Cyclic voltammetry
EDLCs: Electrical double layer capacitors
EIS: Electrochemical impedance spectroscopy
FESEM: Field emission scanning electron microscopy
GCD: Galvanostatic charge-discharge measurements
HRTEM: High- resolution transmission electron microscopy
SAED: Selected-area electron diffraction
SCs: Supercapacitors
SEM: Scanning electron microscopy
TEM: Transmission electron microscopy
XPS: X-ray photoelectron spectroscopy
XRD: X-ray diffraction
